# Total Phenolic Content and Antioxidant and Antimicrobial Activities of *Papaver rhoeas* L. Organ Extracts Growing in Taounate Region, Morocco

**DOI:** 10.3390/molecules27030854

**Published:** 2022-01-27

**Authors:** Anouar Hmamou, Noureddine Eloutassi, Samar Zuhair Alshawwa, Omkulthom Al kamaly, Mohammed Kara, Ahmed Bendaoud, El-Mehdi El-Assri, Sara Tlemcani, Mostafa El Khomsi, Amal Lahkimi

**Affiliations:** 1 Engineering Laboratory of Organometallic, Molecular Materials and Environment (LIMOME), Faculty of Sciences Dhar El Mahraz, Sidi Mohamed Ben Abdellah University, B.P. 1796 Atlas, Fez 30000, Morocco; eloutassinoureddine@gmail.com (N.E.); ahmed.bendaoud@usmba.ac.ma (A.B.); sara.tlemcani@usmba.ac.ma (S.T.); amal.lahkimi@usmba.ac.ma (A.L.); 2 Department of Pharmaceutical Sciences, College of Pharmacy, Princess Nourah bint Abdulrahman University, P.O. Box 84428, Riyadh 11671, Saudi Arabia; SZAlshawwa@pnu.edu.sa (S.Z.A.); omalkmali@pnu.edu.sa (O.A.k.); 3 Laboratory of Biotechnology, Conservation and Valorisation of Natural Resources (LBCVNR), Department of Biology, Faculty of Science Dhar El Mahraz, Sidi Mohamed Ben Abdellah University, B.P. 1796 Atlas, Fez 30000, Morocco; 4 Laboratory of Biotechnology, Environment, Agri-Food and Health, Faculty of Sciences Dhar El Mahraz, Sidi Mohammed Ben Abdellah University, B.P. 1796 Atlas, Fez 30000, Morocco; elmehdi.elassri@usmba.ac.ma; 5 Natural Resources and Sustainable Development Laboratory, Department of Biology, Faculty of Sciences, Ibn Tofail University, B.P. 133, Kenitra 14000, Morocco; elkhomsi.mostafa@uit.ac.ma

**Keywords:** *Papaver rhoeas* L., total polyphenol content, total flavonoid content, antioxidant activity, antimicrobial activity

## Abstract

The objective of this study is to valorize *Papaver rhoeas* L. from the Taounate region of Morocco by determining the total polyphenol content (TPC), the total flavonoid content (TFC) and the antioxidant and antimicrobial activities of four organs. The quantification of TPC and TFC in root, stem, leaf and flower extracts (RE, SE, LE and FE, respectively) was estimated by the Folin–Ciocalteu reaction and the aluminum trichloride method, respectively. Two tests were used to assess antioxidant power: the DPPH test and TAC assay. The antimicrobial activity was studied against five pathogenic bacteria and yeast, using two methods: disk diffusion and microdilution. The TPC in LE and LF was twice as high as that in RE and SE (24.24 and 22.10 mg GAE/g, respectively). The TFC values in the four extracts were very close and varied between 4.50 mg QE/g in the FE and 4.38 mg QE/g in the RE. The LE and FE showed low DPPH values with IC50 = 0.50 and 0.52 mg/mL, respectively. The TAC measurement revealed the presence of a significant amount of antioxidants in the studied extracts, mainly in LE and FE (6.60 and 5.53 mg AAE/g, respectively). The antimicrobial activity results revealed significant activity on almost all of the tested strains. The MIC of FE and SE against *E. coli* 57 was 1.56 and 0.78 mg/mL, respectively, while against the *S. aureus* it was 50 and 25 mg/mL, respectively. The low MLC value (1.56 mg/mL) was recorded against *E. coli* 57 by RE and SE.

## 1. Introduction

Antimicrobial resistance is a difficult condition in which bacteria and fungi develop techniques to reject medications intended to kill them and as a result, germs that are not killed continue to multiply as strongly, if not more so, than before [1]. In recent decades, antibiotic resistance has been regarded as one of the most serious risks to human health and the World Health Organization has listed it as the ninth most severe threat for 2019 [2,3]. Many factors contribute to the emergence of antimicrobial resistance, including the inappropriate use of antibiotics in human medicine, animal husbandry, sanitation and the food sector [4,5]. Aromatic and medicinal plants are the most essential means of treating a variety of antimicrobial illnesses. They contain a variety of antimicrobial chemicals and have no adverse effects [6,7,8]. Furthermore, herbal medicines remain an essential source of treatment for serious diseases, particularly in underdeveloped countries, and 60–80% of the world’s population still uses traditional medicines to treat common ailments [9,10]. In this context, and as part of preliminary research to find active substances in medicinal plants, we chose *Papaver rhoeas* L. (corn poppy), a wild plant known for its use in national and international traditional pharmacopoeias, primarily to aid sleep, as a laxative, to alleviate chest pain and to alleviate the inflammation of the throat and tongue. Furthermore, antipyretic, anti-fever and liver-cooling actions have been reported [11,12].

The corn poppy is a cosmopolitan weed. The exact origin of this plant is not well known. However, some researchers believe that it is native to North Africa, Europe and Western Asia. It is therefore likely that it originates from the eastern Mediterranean basin, as this region connects the three geographical areas mentioned above [13]. The scarlet blossoms and whitish hairs that cover the *Papaver rhoeas* make it easy to identify. It is an erect herb that grows to a height of around 20–80 cm and emits a pungent odor and white latex when damaged [14]. The presence of anthocyanins, of which cyanidol is the main component, gives poppy petals their red color [11].

Several international phytochemical studies show that *Papaver rhoeas* contains a high concentration of secondary and primary metabolites, including amino acids, carbohydrates, fatty acids, vitamins, phenolic compounds, essential oils, flavonoids, alkaloids, coumarins, organic acids and other compounds, which explains its use in nutrition and traditional pharmacopeia [11].

Several pharmacological properties of *Papaver rhoeas* extracts have been reported, including: antidepressant [15], antimicrobial [16,17], antioxidant [18,19], antiulcerogenic [20], cytotoxic, genotoxic and antioxidant [21], morphine-induced CP [22] and sedative [23].

The aim of this investigation is to evaluate and compare the dosage of TPC and TFC, as well as their antioxidant and antimicrobial activities, between the four parts of the *P. rhoeas* plant (root, stem, leaf and flower) of the Taounate region, Morocco.

## 2. Results and Discussion

### 2.1. Extract Yields

The yield of *P. rhoeas* extracts is shown in Table 1 below. The yield of the extracts varied between 18.77% for the LE and 11.77% for the SE. According to [24], the yield of flower extract is 21%. This value remains higher than our results.

### 2.2. Determination of Total Polyphenol Content (TPC)

The TPC in the RE, SE, LE and FE of *P. rhoeas* was carried out using Folin-Ciocalteu assay at 765 nm and the outcomes were expressed as 1 milligram of gallic acid equivalent per 1 g of extract (mg GAE/g of extract), using the following equation of the linear regression of the calibration curve plotted for gallic acid (R^2^ = 0.9948). The results presented in Table 2 show that the average TPC in the RE, SE, LE and FE of *P. rhoeas* is as follows: (10.229 ± 0.183 mg GAE/g, 10.585 ± 0.980 mg GAE/g, 24.240 ± 4.960 mg GAE/g and 22.100 ± 2.220 mg GAE/g, respectively). The results show that LE and FE contain twice the amount of TPC compared with stem and root extracts. The difference is statistically significant (*p* < 0.05). In comparison with the result of other studies, our TPC values in LE and FE are higher than those of the hydro-ethanolic extract of fresh petals of Serbian *P. rhoeas* (14.30 mg GAE/g fresh petals) [25]. In addition, approximately similar values to our LE and FE results were found in the methanolic extract of basal leaves of Spanish *P. rhoeas* (25.86 mg GAE/g extract) [26]. Another Turkish study shows that the TPC in the hydro-ethanolic leaf extract of *P. rhoeas* is 100 mg GAE/g extract, while the TPC in acetone extract is 78 mg GAE/g extract [18]. In Morocco, a recent research shows that the value of TPC in the hydro-ethanolic extract of the dried pollen of *P. rhoeas* is 34.8 mg/g pollen [27]. The obtained TPC values in the extracts from the latter two studies are higher than our results.

### 2.3. Determination of Total Flavonoid Content (TFC)

The TFC in *P. rhoeas* RE, SE, LE and FE was measured using reagent aluminum trichloride (AlCl_3_) assays at 430 nm and the result were represented in milligrams of quercetin equivalent per 1 g of extract (mg QE/g extract) using the equation (R^2^ = 0.99). the results presented in Table 2 show that the average content of total flavonoids in the RE, SE, LE and FE of *P. rhoeas* is as follows: (4.381 ± 0.090 mg QE/g, 4.493 ± 0.082 mg QE/g, 4.391 ± 0.075 mg QE/g and 4.500 ± 0.072 mg QE/g, respectively). The difference in TFC between the four *P. rhoeas* parts is not statistically significant (*p* < 0.05). When compared to previous literature, a recent Moroccan study found that the TFC in the methanolic extract of *P. rhoeas* flowers extracted by the maceration method is 8.67 mg QE/g of extract [24], as well as in Moroccan hydro-ethanolic dried pollen maceration extract, which shows a value of 12.95 mg QE/g of pollen [27]. In another Serbian study, the TFC in fresh petal extract of *P. rhoeas* is 9.07 mg QE/g fresh petals, using the ultrasound procedure in an aqueous-ethanolic medium [25]. A Spanish study shows that TFC in a methanolic extract of basal leaves of *P. rhoeas* is 12.00 CE mg/g extract [26]. The TFC values in these cited studies are higher than our results obtained. In 2004, ref. [28] conducted a phytochemical investigation that revealed the presence of the flavonoids kaempferol, quercetin, hypolaetin and luteolin, as well as the flavonoid glycosides isoquercitrin, astragalin and hyperoside in methanolic extracts.

The variability of TPC and TFC could be due to the climatic conditions, geographical location, fertility of the soil, genotype of cultivar and experimental factors, as the plant part used, harvesting time, method of extraction and polarity of solvent used, as well as the length of the extraction [29,30].

### 2.4. Determination of Antioxidant Activity

#### 2.4.1. Scavenging of the Free Radical DPPH

**Figure 1:**
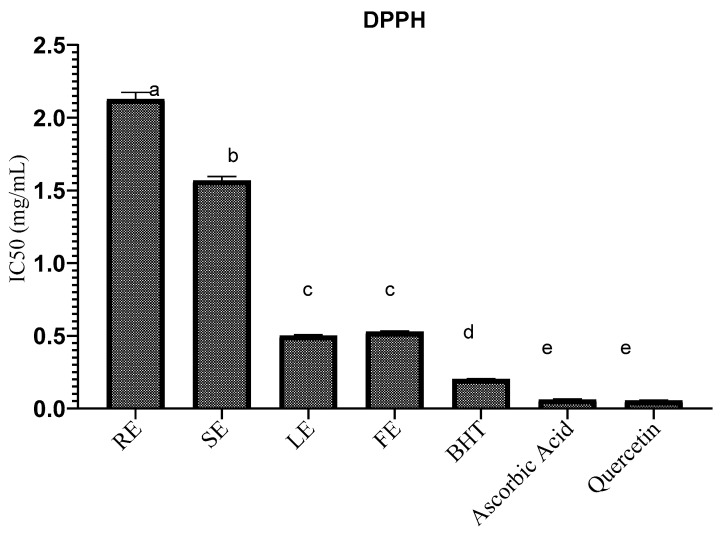
Antiradical activity (IC50) of *Papaver rhoeas* extracts; a, b, c, d and e—values with a significant difference.

The antioxidant activity of the four parts of the extracts of *P. rhoeas* was investigated by the trapping capacity of the free radical DPPH. The outcomes obtained in the test to measure the IC50 are shown in Figure 1. The IC50 value is the effective concentration that requires a 50% reduction in the starting DPPH concentration. A lower IC50 value indicates a more effective protective effect. The IC50 of the RE, SE, LE and FE of this plant are 2.12 ± 0.044 mg/mL, 1.56 ± 0.027 mg/mL, 0.50 ± 0.007 mg/mL and 0.52 ± 0.005 mg/mL, respectively. Compared with the antioxidant activities of reference substances (BHT and quercetin), which are 0.20 ± 0.004 and 0.06 ± 0.004 mg/mL, respectively, the antioxidant activity of the 4 parts of this plant is lower. Except for FE and LE, the difference in IC50 across the 4 part extracts is statistically significant (*p* < 0.05). The antioxidant activity of the LE is higher than that of the FE, followed by the SE and finally the RE. The results obtained show an antioxidant activity much greater than that found in flower extracts (maceration and soxlet) by [24], with an IC50 = 4.97 and 3.81 mg/mL, respectively. Another Turkish study found that the aqueous, ethanolic and acetone extracts of *P. rhoeas* leaves had antioxidant activity, with IC50 values of 1.39 mg/mL, 3.11 mg/mL and 5.49 mg/mL, respectively [18]. Our results remain better in comparison with the results of this study.

#### 2.4.2. Total Antioxidant Capacity (TAC)

**Figure 2:**
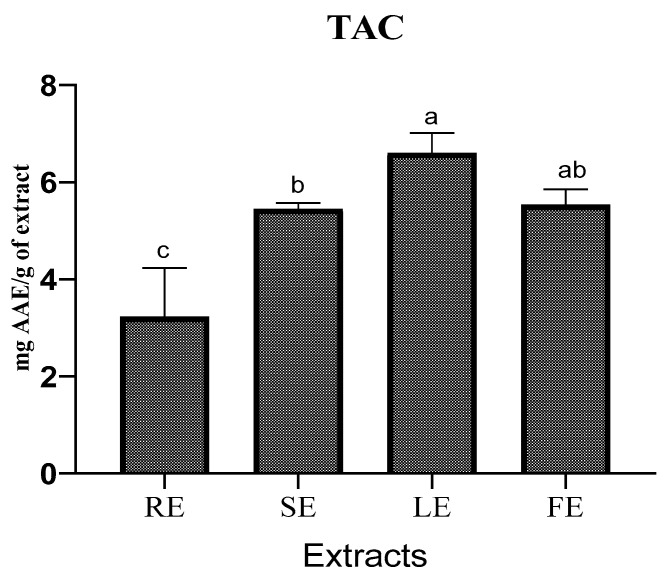
TAC of *P. rhoeas* extracts. a, b and c—values with a significant difference.

The TAC is measured in milligrams of ascorbic acid equivalent per one gram of extract (mg AAE/g) in this investigation, with ascorbic acid serving as the reference ingredient. The results show that the four extracts have different antioxidant activities (Figure 2). The LE has the best TAC of about 6.60 ± 0.414 mg/g of extract, followed by the FE, which has a TAC of about 5.53 ± 0.322 mg/g of extract, followed by the SE, which has a TAC of about 5.45 ± 0.124 mg/g of extract, and finally, the RE has a TAC of 3.22 ± 1.005 mg/g of extract. The difference in TAC between the four extracts of *P. rhoeas* is statistically significant (*p* < 0.05). The TAC test and the DPPH test of the four *P. rhoeas* extracts are in accord. According to these results, the four organs of *P. rhoeas*, particularly the flowers and leaves, can be a natural source of antioxidants. In an Italian study, the TAC of *P. rhoeas* extract was 43.89 mmol TE/g, a value explained by the high phenolic compound content detected [19].

### 2.5. Antimicrobial Activity

#### 2.5.1. Disc Inhibitory Assay

The disc diffusion test was carried out to investigate the antibacterial activity of *P. rhoeas* RE, SE, LE and FE against five pathogen strains. Table 3 summarizes the results of measuring the diameter of the inhibitory zone (DIZ). Generally, the bulk of the strains were sensitive to our extracts. The DIZ values for *P. rhoeas* extracts varied from 13.66 ± 0.57 mm against *Staphylococcus aureus* to 8.33 ± 0.57 mm against *Klebsiella pneumoniae*. The stem and flower extracts had no effect on the *Klebsiella pneumoniae* strain, while all of the extracts from this plant had no effect on *Candida albicans* yeast. The DIZ value between *P. rhoeas* extracts is statistically significant (*p* < 0.05) against *Escherichia coli* 97 and *Staphylococcus aureus*, but not significant against *Escherichia coli* 57 and *Klebsiella pneumoniae*. When compared with the use of standard antibiotics, we found that all pathogenic bacterial strains were resistant to the streptomycin and ampicillin used, with the exception of *Staphylococcus aureus*, which was sensitive to streptomycin with a DIZ of 9.61 ± 0.20 mm. While the application of fluconazole to the yeast *Candida albicans* gave a zone of inhibition of 21.20 ± 04.200 mm. In a Moroccan study, the values of the DIZ of *P. rhoeas* aerial parts extract from the Sidi Bennour region against the pathogenic strains *S. aureus*, *E. coli* and *C. albicans* were 8 ± 2.00 mm, 6 ± 2.08 mm and 6 ± 2.51 mm, respectively [31]. Our results remain the best in comparison with this study.

#### 2.5.2. Determination of Minimum Inhibitory Concentration (MIC) and Minimum Lethal Concentration (MLC) of *P. rhoeas* Extracts

According to the results of DIZ, it was observed that *P*. *rhoeas* RE, SE, LE and FE affected most of the studied microorganisms. The minimal inhibitory concentration (MIC) values of the extracts were evaluated against most bacteria strains. The results are shown in Table 4. The MIC of FE and SE against *E. coli* 57 (G−) was 1.56 mg/mL and 0.78 mg/mL, while for the *S. aureus* (G+), the MIC was 50 and 25 mg/mL, respectively. Unlike the disk diffusion test, which shows no activity of all *P.* extracts against the yeast *C. albicans*, the microdilution method shows only the stem extract at an MIC of 12 mg/mL against this yeast. The antibiotics streptomycin and fluconazole have a lower MIC than our studied extracts, while ampicillin has no activity. Comparing our results with the other studies shows that our results are also consistent with the literature regarding the antimicrobial activity of *P. rhoeas* extract against all microbial strains tested. The *P. rhoeas* ethanolic extracts of Turkey show antibacterial effect against the *S. aureus* strain with an MIC of 0.15 mg/mL and no activity against the strains *E. coli*, *K. pneumoniae* and *C. albicans.* In addition, diethyl ether, chloroform and acetone extracts from the same plant against *S. aureus* showed significant antimicrobial (MIC = 39.06 μg/Ml) [17]. In another study, the chloroform extract of the aerial parts of *P. rhoeas* showed the most significant effect *S. aureus* (MIC = 1.22 μg/mL), and had high antimicrobial action against *K. pneumoniae* and *S. epidermidis*. There was no action against *P. mirabilis* [16]. In a Moroccan study, the MIC values of the antibacterial activities in vitro of flower extract of *P. rhoeas* against pathogenic bacterial strains (*E. coli*, *K. pneumoniae* and *S. aureus*) were 60 mg/mL, 30 mg/mL and 60 mg/mL, respectively [24].

The MLC is the minimum antibacterial concentration necessary to kill a certain bacterium [32]. The MLC concentrations of four extracts ranged from 1.56 mg/mL to over 50 mg/mL. The lowest MLC values against *E. coli* (ATB: 57) were 1.56 mg/mL for the RE and SE and 3.12 mg/mL for the SE and FE against *E. coli* 97 and *E. coli* 57, respectively. In general, our results show that the SE has the highest antibacterial activity, while the LE has the lowest antibacterial activity. This difference in antibacterial activity can be explained by the qualitative and quantitative differences between TPC and TFC in the four extracts of *P. rhoeas*.

In this regard, ref. [33] indicated that the strongest antimicrobial activity may be linked to the phenol fraction’s wealth, especially the existence of structural hydroxyl-phenol groups, which add to the rise in antimicrobial impact. Indeed, the treatment of bacteria with phenol substances alters the cell membrane structure, lowers lipid content and ultimately impedes microbial development [34]. By saturating the cell membrane, these elements allegedly damage or enter the lipid structures of cells [35]. Furthermore, ref. [16] discovered a significant relationship between the antibacterial activity of *P. rhoeas* extracts and their alkaloid composition, particularly roemerin, which possesses an aporphine alkaloid skeleton and methylenedioxy fragment.

### 2.6. Correlation between Investigated Quality Parameters of P. rhoeas Extracts

The Pearson’s correlation coefficients between the different parameters studied here revealed a positive correlation between several parameters (Table 5), including *E. coli* 97 and *K. pneumonia* strains (r = 0.955), TFC and *E. coli* strain (r = 0.763) and TPC with TAC and *E. coli* 57 strain (r = 0.748, r = 0.653, respectively). The TAC has a positive correlation with *E.coli *57, *E.coli *97 and *K. pneumonia* strains (r = 0.665, r = 0.858 and r = 0.679, respectively). On the other hand, TPC is strongly negatively correlated with IC50 (r = −0.959) and the latter is negatively correlated with TAC and *E. coli *57 (r = −0.861 and r = −0.813, respectively). A significant association between bioactive substances and free radical scavenging capacity indicates that antioxidants, such as TPC and TFC, have a significant contribution on the free radical scavenging capability of *P. rhoeas*. Several investigations have demonstrated that increasing overall phenolic content is related to better free radical scavenging activity and that there is a linear association between phytochemical compounds and antioxidant activity [36,37,38].

**Figure 3:**
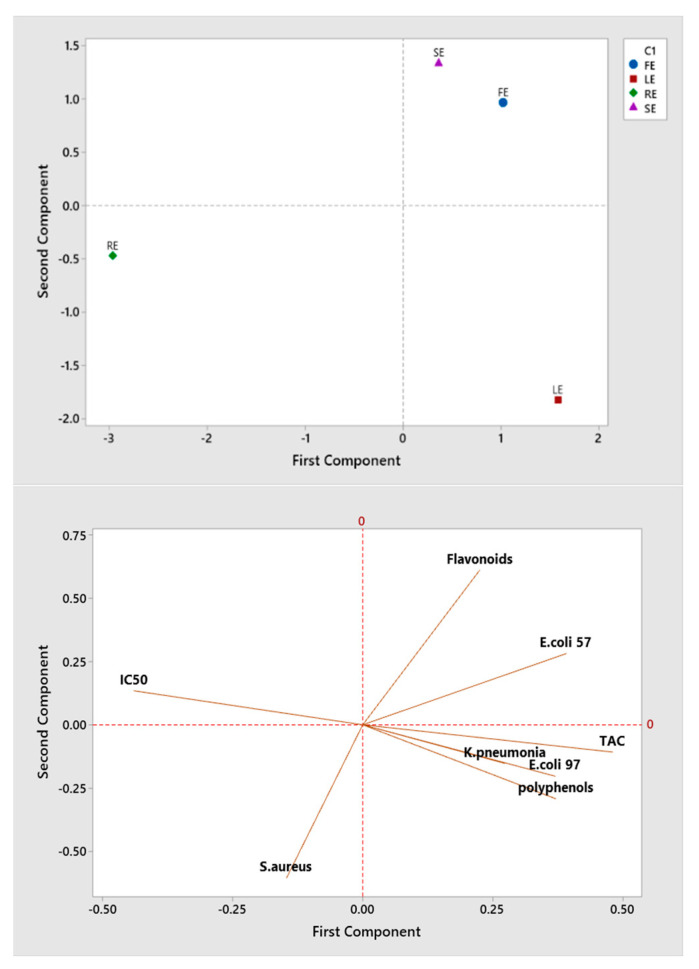
Principal component analysis of different studied parameters.

The principal component analysis (PCA) obtained, shown in Figure 3, shows that the eigenvalues of the first 2 principal components represent 78.1% of the variation in the data.

The projection of the scoring diagram and the contribution diagram visually shows a positive contribution of polyphenols, TAC, *E. coli* 57 and *E. coli* 97 on the first main axis in negative correlation with RE. However, the flavonoids and *S. aureus* contributed on the second main axis in correlation with SE and FE.

## 3. Materials and Methods

### 3.1. Plant Material

The *P. rhoeas* plants were harvested at the end of April 2021 from the Tissa region (36 km from Taounate city). The plants were identified by the botanist Amina Bari, professor in the Faculty of Sciences at Dhar El Mahraz, Sidi Mohamed Ben Abdellah University, Fez, Morocco. The four parts of *P. rhoeas* were carefully washed with distilled water, then left to dry in the laboratory in the absence of light, humidity and dust. After a while, each plant organ was crushed using a grinder and stored in a small, tightly closed and labeled glass vial.

### 3.2. Extracts Preparation

The powder (20 g) of the 4 dried organs was extracted by hydro-ethanolic maceration consisting of 70% ethanol and 30% distilled water for 48 h at room temperature. The extraction product has been filtered using a Whatman filter with a porosity of 0.22 mm. Rotary evaporation was used to remove the solvent from the fraction. Until it was used, the finished product was kept at 4 degrees Celsius. The extraction yield of four *P. rhoeas* parts was calculated in relation to the total dry matter according to the following relationship:

Y (%) = (ME/DM) × 100(1)

Y—yield of extract in %; ME—mass of extract collected in g; DM—dry matter in g.

### 3.3. Determination of Total Phenols Content (TPC)

The TPC of *P. rhoeas* RE, SE, EL and EF were tested by the Folin–Ciocalteu method described by [39]. Briefly, (200 μL) of each extract is added to (1 mL) of Folin-Ciocalteu (10%). After 4 min of incubation, (800 μL) of sodium carbonate (7.5%) is added. A UV-Vis spectrophotometer was used to measure the absorbance at 765 nm after the mixture had been incubated at room temperature for 2 h. The TPC was determined using a standard curve established with gallic acid as the reference. The result was expressed in milligrams of gallic acid equivalent per gram of extract (mg GAE/g of extract). All experiences are carried out in triplicate.

### 3.4. Total Flavonoids Content (TFC)

The TFC was estimated by the AlCl_3_ method [40]. Briefly, the hydro-ethanolic extracts of each part of *P. Rhoeas* (1 mL) were combined with (1 mL) aluminum trichloride (AlCl_3_, 2%) methanolic solution. The absorbance was determined at 430 nm against a blank (methanol solution) after 15 min. The reference compound was quercetin (positive blank). The results were given in milligrams of quercetin equivalents per gram of extract (mg QE/g of extract). All tests are carried out three times.

### 3.5. Antioxidant Activity

#### 3.5.1. Antioxidant Activity by the Free Radical DPPH

The determination of antioxidant activity by the DPPH radical scavenging method was realized according to the protocol described by [41]. Briefly, (100 μL) of each extract methanol solution was added to 750 μL DPPH in methanol (0.004%) at various concentrations. The absorbance is measured using a spectrophotometer at 517 nm after 30 min of incubation at room temperature. A negative blank was prepared by mixing 0.75 mL of DPPH solution with 100 μL of methanol. Each test was realized in triplicate. The use of the following relationship allowed us to estimate the antioxidant activity of the extracts:

% antiradical activity = (1 − (*A*1/*A*0)) × 100 (2)

where *A*0 is the absorbance of a negative control (blank sample containing the same amount of solvent and DPPH solution) and *A*1 is the sample absorbance. The % antiradical activity (IC50) values may be visually calculated using linear regression, revealing the inhibitory concentration of the diphenyl picryl-hydrazyl radical (DPPH) at 50%.

#### 3.5.2. Total Antioxidant Capacity Test (TAC)

A measure of one mL of reagent solution (28 mM sodium phosphate, 0.6 M sulfuric acid and 4 mM ammonium molybdate) was added to 25 μL of each extract. After that, the solution was incubated for 90 min at 95 degrees Celsius. The resulting solution’s optical density was determined at 695 nm. The extract’s TAC was calculated using milligrams of ascorbic acid equivalent per gram of extract (mg AAE/g) [42]. The test was then repeated three times.

### 3.6. Antimicrobial Activity Evaluation

In this work, the antibacterial activity of *P. rhoeas* RE, SE, EL and EF was evaluated against four pathogenic bacteria and one pathogenic yeast, all of which are responsible for many illnesses. Both *Escherichia coli* (ATB: 97) BGM (Gram−) and *Escherichia coli* (ATB: 57) B6N (Gram−) were taken from the Hassan II University Hospital of Fez, while *Klebsiella pneumonia* (Gram−), *Pseudomonas aeruginosa* (Gram−), *Staphylococcus aureus* (Gram+) and *Candida albicans* were obtained from the laboratory of microbiology, Faculty of Medicine and Pharmacy, Fez, and all were used as test microorganisms. The microbial cultures were kept in the refrigerator (4 °C) on Mueller–Hinton (MH) agar.

#### 3.6.1. Tested Strains and Inoculum Standardization

To get a young culture and single colonies, the different microbial strains were streaked into Petri dish Mueller-Hinton agar and incubated at 37 °C for 18–24 h. Each strain’s isolated colony was levied with a platinum loop, homogenized in sterile saline (NaCl, 0.9%) and pre-cultured at 37 °C for 3–5 h before being adjusted to the turbidity 0.5 McFarland (corresponding to 1–5 × 10^8^ CFU/mL) [43].

#### 3.6.2. Disc Diffusion Method

The bacterial strains were lawn cultured using an autoclaved cotton swab soaked in a standardized solution (1–5 × 10^8^ CFU/mL) and wiped over the MHA plate surface. Subsequently, the discs of paper Whatman (6 mm) were put on the surface of pre-inoculated agar and impregnated with 10 μL of the tested extracts. The ampicillin 1.67 mg/disc (AMP antibiotic), streptomycin 0.02 mg/disc (STR antibiotic) and fluconazole 5 mg/disc (FLU antifungal) were used as a positive control. The widths of the inhibitory zones were determined after incubation at 37 °C during 24 h. All of these tests were repeated three times to acquire the average value of inhibition zone, which was then used to compute the standard deviation [44].

#### 3.6.3. Determination of Minimum Inhibitory Concentration (MIC)

The MICs of RE, SE, EL and EF were determined using the microdilution method in 96-well microplates, with minor modifications, according to NCCLS standards [45]. Our extracts were disposed of in sterile tubes at 10 different concentrations, which were achieved by a series of 1/2 dilutions in distilled water. In each series of microplate wells, the range of concentrations of RE, SE, EL and EF were from 0.975 to 50 mg/mL. The microbial suspensions were prepared as reported above [43]. Briefly, twenty microliter of each preparation were diluted in 80 μL of liquid MH broth and plated at a density of 50 × 10^5^ CFU/well in 96-well plates. Finally, 100 μL of various concentrations of our extracts were applied to each well except the last well (positive growth control) to determine the MIC values. The colorimetric approach was carried out using the dye reagent (triphenyltetrazoluim chloride (TTC)) after 24 h of incubation at 37 °C. The MIC values were determined after 2 h of incubation as the lowest concentration that did not cause pink color and had a high capacity to detect strain development in the wells [46].

#### 3.6.4. Minimum Lethal Concentration (MLC)

With slight adjustments, the MLC values were determined as specified in document M26-A [47]. Three wells were obtained using a cotton swab and compared to the MICs. The number of viable cells (CFU/mL) must be assessed after they have grown on the surface of the non-selective agar plate. The lethal endpoint (MLC) is considered as a concentration that can kill 99.9% of the final inoculum [48].

### 3.7. Statistical Analysis

The mean and standard deviations (SD) of the data were calculated. The data were analyzed using a one-way ANOVA, with *p* < 0.05 indicating a significant difference between means, as determined by a multiple range test utilizing the least significant difference (LSD) or Duncan’s test at α < 0.05. Multiple correspondence analysis was carried out to evaluate the homogenous grouping.

## 4. Conclusions

In the present work, we investigated the chemical composition, antioxidant capacity and antimicrobial activity of four parts of *P. rhoeas* from the Taounate region, Morocco. The results of this study show that the four parts (root, stem, leaf and flower) of *P. rhoeas* are rich in TPC and TFC, mainly the leaves and flowers. The four parts show very significant antioxidant and antimicrobial activities. The leaves and flowers have the highest antioxidant activity, while the stem has the highest antimicrobial activity. Previously, the whole plant of *P. rhoeas* was considered biomass waste, but after this study, it is necessary to exploit the antimicrobial properties of this plant in the pharmaceutical field to make antibiotics based on natural molecules to fight against antibiotic-resistant strains. Additionally, it is necessary to exploit the antioxidant properties of this plant in the agri-food as a natural antioxidant for the preservation of food without any negative risk to human health.

## Figures and Tables

**Table 1:** Extraction yield of *P. rhoeas* extracts.

Sample	Mass of Dry Matter (g)	Mass of The Extract (g)	Yield (%)
RE	20	2.46	12.30
SE	20.84	2.35	11.77
LE	20.20	3.75	18.77
FE	20.70	3.72	18.60

RE—root extract; SE—stem extract; LE—leaf extract; FE—flower extract.

**Table 2:** Total polyphenol contents and total flavonoid contents in the *P. rhoeas* extracts.

Sample	TPC (mg GAE/g of Extract)	TFC (mg QE/g of Extract)
RE	10.229 ± 0.183 ^b^	4.381 ± 0.090 ^a^
SE	10.585 ± 0.980 ^b^	4.493 ± 0.082 ^a^
LE	24.240 ± 4.960 ^a^	4.391 ± 0.075 ^a^
FE	22.100 ± 2.220 ^a^	4.500 ± 0.072 ^a^

RE—root extract; SE—stem extract; LE—leaf extract; FE—flower extract; a and b—values with a significant difference.

**Table 3:** Diameter of the inhibition zone of *P. rhoeas* extracts and antibiotics (mm).

Sample/Antibiotic	Gram-Negative Bacteria			Gram-Positive Bacteria	Yeast
	*E. coli* 57	*E. coli* 97	*K. pneumoniae*	*S. aureus*	*C. albicans*
RE	12.66 ± 1.15 ^a^	12.00 ± 0.00 ^b^	ND	13.66 ± 0.57 ^a^	R
SE	13.00 ± 1.00 ^a^	13.00 ± 0.00 ^ab^	8.33 ± 0.57 ^a^	11.00 ± 0.00 ^b^	R
LE	13.00 ± 0.00 ^a^	13.33 ± 0.57 ^a^	8.67 ± 1.15 ^a^	13.66 ± 0.57 ^a^	R
FE	13.33 ± 1.52 ^a^	12.33 ± 0.57 ^ab^	ND	12.33 ± 0.57 ^c^	R
Streptomycin	R	R	R	9.61 ± 0.20	---
Ampicillin	R	R	R	R	---
Fluconazole	---	---	---	---	21.20 ± 04.20

R—resistant; ND—none detected; “---“—antibiotic does not match this strain; a, b and c—values with a significant difference.

**Table 4:** Minimal inhibitory concentration (MIC) and minimum lethal concentration (MLC) of *P. rhoeas* extracts (mg/mL).

Sample	Gram-Negative Bacteria						Gram-Positive Bacteria		Yeast	
	*E. coli* 57		*E. coli* 97		*K. pneumoniae*		*S. aureus*		*C. albicans*	
	MIC	MLC	MIC	MLC	MIC	MLC	MIC	MLC	MIC	MLC
RE	0.78	1.56	3.12	6.25	ND	ND	25	25	ND	ND
SE	0.78	1.56	1.56	3.12	6.25	6.25	6.25	12.5	12	12.5
LE	50	>50	50	>50	ND	ND	50	>50	ND	ND
FE	1.56	3.12	3.12	6.25	ND	ND	50	>50	ND	ND
Streptomycin	0.25		0.50		0.003		0.062		---	
Ampicillin	R		R		R		R		---	
Fluconazole	---		---		---		---		0.40	

R—resistant; ND—none detected; “---“—antibiotic does not match this strain.

**Table 5:** Pearson correlation coefficients between different parameters of *Papaver rhoeas*.

	TPC	TFC	IC50	TAC	*E. coli* 57	*E. coli* 97	*S. aureus*
TFC	0.014						
IC50	−0.959	−0.272					
TAC	0.748	0.302	−0.861				
*E. coli* 57	0.653	0.763	−0.813	0.665			
*E. coli* 97	0.406	0.040	−0.503	0.858	0.224		
*S. aureus*	0.333	−0.881	−0.052	−0.203	−0.426	−0.191	
*K. pneumonia*	0.118	−0.003	−0.226	0.679	0.000	0.955	**−0.278**

## Data Availability

Not applicable.

## Sample Availability

Samples of the compounds are not available from the authors.
